# Excess Mortality Associated With Loiasis: Confirmation by a New Retrospective Cohort Study Conducted in the Republic of Congo

**DOI:** 10.1093/ofid/ofad103

**Published:** 2023-02-24

**Authors:** Marlhand C Hemilembolo, Ange Clauvel Niama, Jérémy T Campillo, Sébastien D Pion, François Missamou, Charles Whittaker, Jean-Médard Kankou, Gilbert Ndziessi, Richard R Bileckot, Michel Boussinesq, Cédric B Chesnais

**Affiliations:** UMI 233 TransVIHMI, Université de Montpellier, Institut de Recherche pour le Développement (IRD), INSERM Unité, Montpellier, France; Programme National de Lutte contre l'Onchocercose, Direction de l'Épidémiologie et de la Lutte contre les Maladies, Ministère de la Santé et de la Population, Brazzaville, République du Congo; MRC Centre for Global Infectious Disease Analysis, Jameel Institute for Disease and Emergency Analytics, Imperial College London, London, United Kingdom; UMI 233 TransVIHMI, Université de Montpellier, Institut de Recherche pour le Développement (IRD), INSERM Unité, Montpellier, France; UMI 233 TransVIHMI, Université de Montpellier, Institut de Recherche pour le Développement (IRD), INSERM Unité, Montpellier, France; Programme National de Lutte contre l'Onchocercose, Direction de l'Épidémiologie et de la Lutte contre les Maladies, Ministère de la Santé et de la Population, Brazzaville, République du Congo; MRC Centre for Global Infectious Disease Analysis, Jameel Institute for Disease and Emergency Analytics, Imperial College London, London, United Kingdom; Programme National de Lutte contre l'Onchocercose, Direction de l'Épidémiologie et de la Lutte contre les Maladies, Ministère de la Santé et de la Population, Brazzaville, République du Congo; Faculté des Sciences de la Santé, Université Marien-Ngouabi, Brazzaville, République du Congo; Faculté des Sciences de la Santé, Université Marien-Ngouabi, Brazzaville, République du Congo; UMI 233 TransVIHMI, Université de Montpellier, Institut de Recherche pour le Développement (IRD), INSERM Unité, Montpellier, France; UMI 233 TransVIHMI, Université de Montpellier, Institut de Recherche pour le Développement (IRD), INSERM Unité, Montpellier, France

**Keywords:** loiasis, Republic of Congo, cohort study, filariasis, mortality

## Abstract

**Background:**

Loiasis (*Loa loa* filariasis) is considered a benign disease and is currently not included in the World Health Organization’s (WHO's) list of Neglected Tropical Diseases, despite mounting evidence suggesting significant disease burden in endemic areas. We conducted a retrospective cohort study to assess the mortality associated with *L. loa* microfilaremia in the Southwestern Republic of Congo.

**Methods:**

The cohort included 3329 individuals from 53 villages screened for loiasis in 2004. We compared mortality rates in 2021 for individuals initially diagnosed as with or without *L. loa* microfilariae 17 years earlier. Data were analyzed at the community level to calculate crude mortality rates. Survival models were used to estimate the effect of *L. loa* microfilaremia on mortality in the population.

**Results:**

At baseline, prevalence of microfilaremia was 16.2%. During 17.62 years of cohort follow-up, 751 deaths were recorded, representing a crude mortality rate of 15.36 (95% CI, 14.28–16.50) per 1000 person-years. Median survival time was 58.5 (95% CI, 49.7–67.3) years and 39.2 (95% CI, 32.6–45.8) years for amicrofilaremic and microfilaremic indiviudals, respectively.

**Conclusions:**

A significant reduction in life expectancy was associated with *L. loa* microfilaremia, confirming previous observations from Cameroon. This adds to the evidence that loiasis is not a benign disease and deserves to be included in the WHO's list of Neglected Tropical Diseases.

Loiasis is a parasitic disease caused by the filarial worm *Loa loa* and transmitted from human to human by bites of tabanid flies (mainly *Chrysops silacea* and *C. dimidiata*). The adult parasites live under the skin or in the intermuscular fascia, while the embryos, called microfilariae (mf), circulate in the peripheral blood. *L. loa* is endemic only in Central Africa, primarily in forested areas. In 2015, it was estimated that 5 million people had *L. loa* mf in their blood [1]—this represents only 35% to 60% of the total population infected with the adult stage due to the existence of “occult” loiasis (infection without microfilaremia) [2, 3]. In 2011, it was estimated that 14 million people lived in areas where loiasis was hyperendemic, that is, where >40% of the population had a history of subconjunctival migration of an adult worm (“eyeworm,” a typical manifestation of loiasis) [4].

Despite its widespread geographic distribution and high prevalence in some settings, interest in loiasis is primarily due to its impact on onchocerciasis control/elimination programs in Central Africa. The presence of loiasis complicates control programs using ivermectin against onchocerciasis [5] because of the risk of potentially fatal post-treatment encephalopathy in individuals with high *L. loa* microfilarial densities (MFDs) [6–8]. However, beyond this, loiasis is also a major reason for medical consultation in endemic areas due to its common manifestations (pruritus, eyeworm, and transient edema called “Calabar swellings”) [2, 9]. Additionally, many reports suggest that loiasis may be associated with renal, splenic, and possibly cardiac impairment, the frequency of which remains unclear [10–12]. A meta-analysis of loiasis case reports showed that the frequency of “atypical loiasis” (ie, with neurological, cardiac, and/or renal complications) increased significantly with *L. loa* MFDs [11], while a cohort study conducted in 2016 in Cameroon showed that individuals with very high *L. loa* MFDs had a significantly reduced life expectancy [13].

Here, we present the results of a retrospective cohort study, following the same methodology used in the study performed in Cameroon [13], which was conducted in 2021 in the Republic of Congo to verify whether the loiasis-associated excess mortality observed in Cameroon existed also in this country. In this study, we evaluated the survival of individuals whose *L. loa* MFDs had been measured in 2004.

## METHODS

### Study Sites and Population

The data collected in March 2004 aimed at validating a rapid assessment method to assess loiasis endemicity levels [4]. This method (called RAPLOA) was developed in 2001 across sites in Cameroon and Nigeria [4, 14, 15]. In 2004, additional studies were conducted in sites across the Democratic Republic of Congo [16], as well as 3 departments of the Republic of Congo (Lékoumou, Bouenza and Niari), to validate the method based on a questionnaire on the participants’ history of eyeworm episodes. In the Republic of Congo, the RAPLOA 2004 database included data from 3329 individuals aged ≥15 years living in 53 villages (age ≥15 years being the only inclusion criteria in 2004). The RAPLOA questionnaire was administered to all participants, who also underwent a fingerprick blood sample to prepare a thick blood smear aimed at measuring their *L. loa* MFD.

The 53 villages were located in 3 environmentally distinct zones: a dense forest zone, a dry savannah/grassland zone, and a mixed zone with gallery forests. Parasitological indicators of loiasis recorded in these villages in 2004 are presented in Supplementary Tables 1–3. These villages were all located in the southwest of the Republic of Congo in areas where onchocerciasis is hypo-endemic or nonendemic and had therefore never received community-directed treatment with ivermectin (CDTI).

### Exposure and Outcome Definition

The main exposure factor was defined as *L. loa* microfilaremic status (positive vs negative) in 2004. We also conducted analyses using *L. loa* MFD categories. In the first, we used 4 categories: MFD = 0 and, for the microfilaremics, 3 categories balanced for sample size. In the second, we considered 3 categories: MFD = 0; 1 < MFD <10 000 mf/mL; and MFD ≥10 000 mf/mL).

In November 2021, 17.62 years after the first survey, we returned to the 53 villages to obtain information on the vital status (dead vs alive) of the 3329 cohort subjects. The information was collected with the assistance of the village committees, relatives of the participants, or the participants themselves. Information on the date of death was collected if the person was deceased. A calendar with major local events was used to determine the dates of death accurately. If only the year was known, the middle of the year (1st of July) was defined as the date of death. If the month was known without the day, the middle of the month (15th day) was assigned.

Dates of the latest news were collected for subjects lost to follow-up (LTFU), that is, individuals who migrated out of their 2004 village of residence and for whom the vital status at the time of the 2021 survey was not known. Dates of the latest news for which the exact day was not known were collected using the same procedure as for dates of death. The follow-up of individuals in the statistical analysis was then censored from these dates. Finally, if no information on dates was available (ie, for those LTFUs with no information available or for persons unknown to the population), the follow-up data were censored halfway between the 2 surveys.

### Covariates

The selected covariates were age (continuous), sex, presence/absence of *Mansonella perstans* (another filarial species) mf, history of eyeworm episodes, village environment (forest, mixed, or savannah), and the community microfilarial load (CMFL) of *L. loa* for each village, divided into 4 balanced classes. The CMFL uses the geometric mean of the individual MFDs (mf/mL), so all MFDs with value 0 were assigned the value of 1 to facilitate their inclusion in the calculation.

The survey was approved by the Institutional Ethics Committee of the Congolese Foundation for Medical Research (No. 032/CIE/FCRM/2021) and the Ministry of Health and Population (authorization 00417/MSP/IGS-21).

## Data Analysis

### Estimation of Crude Mortality Rates

Crude mortality rates (CMRs) were calculated by dividing the number of individuals who died between March 2004 and November 2021 by the number of person-years of follow-up (PY) and expressed per 1000 PY, with PY calculated according to the previously estimated dates (survey date for those who were still alive, dates of the latest news for LTFUs, date of death for the deceased). CMRs were calculated for each subcategory of variable, and statistical differences were assessed using the Mantel-Haenszel test.

### Individual-Level Survival Analysis

We evaluated the existence of interactions using the likelihood ratio test, and the proportional hazards hypothesis was tested using the Schoenfeld residuals test. If the latter condition was not met, an accelerated failure time model (AFT model) was used, with the best-fitting distribution (Weibull, log-normal, or log-logit) determined according to the Akaike information criterion. A possible random effect on the villages was evaluated using the likelihood ratio test. The correlations between the variables were assessed with Cramer's V test. Significant variables were selected from our saturated model in applying a step-by-step manual descending procedure using a likelihood ratio test (with *P* < .050). Following model fitting, predicted effects of our main exposure on survival were estimated.

Based on the distribution of *L. loa* MFDs, in addition to our main evaluation (negative vs positive status), we created the following MFD categories: 0 mf (2789 individuals, 83.8% of the cohort subjects), 1–259 mf/mL (182 individuals, 5.5%), 260–1799 mf/mL (179 individuals, 5.4%), and ≥1800 mf/mL (179 individuals, 5.4%). We also created another categorization to evaluate the influence of higher MFDs: 0 mf, 1–9999 mf/mL (474 individuals, 14.2% of the cohort subjects), and ≥10 000 mf/mL (66 individuals, 2.0%).

### Senstivity Analyses

To explore the effect of LTFUs on survival results, we performed 2 sensitivity analyses. In the first, all LFTUs were removed from the analysis. In the second sensitivity analysis, we carried out an average treatment effect (ATE) analysis at the population level using a propensity score–based approach to obtain balanced covariate distributions across exposure statuses (*L. loa*–negative and –positive status) (Supplementary Data).

Descriptive and survival analyses were performed using R software, version 4.1.3. ATE analyses were performed using STATA software (version 17.1; StataCorp, College Station, TX, USA).

## RESULTS

### Prevalence of *L. loa* Microfilaremia in 2004

Five hundred forty of the 3329 participants were microfilaremic for *L. loa* in 2004 (overall prevalence: 16.2%) (Supplementary Table 4). The prevalence was higher in the forest areas (27.4%) than in the mixed (15.8%) and savannah (6.5%) areas (*P* < .001).

### Lost to Follow-up in November 2021

Among the 3329 cohort subjects, 440 (13.2%) were LTFU between 2004 and 2021. These subjects were significantly younger (mean age, 34 years vs 42 years; *P* < .001). The proportion of LTFU was lower in males than in females (11.9% vs 14.4%; *P* = .035) and not significantly different between microfilaremics and amicrofilaremics at baseline (10.7% vs 13.7%; *P* = .073). Among these 440 people, the year and month of last news could be determined for 173 (39.3%), and the year but not the month for 161 (36.6%). The respondants in the village could not provide a year of last news for, or did not know the names of, 106 people (24.1% of the LTFUs).

### Crude Mortality Rate

During 17.62 years of follow-up, 751 deaths (22.6%) were recorded in the cohort (mean follow-up, 14.70 years; 48 865 PY). The overall crude mortality rate was 15.36 (95% CI, 14.28–16.50) per 1000 PY (Table 1).

**Table 1.** Distribution of Specific Mortality Rate by Subgroup and Crude Mortality Rate in the Population

	PY	Deaths	SMR (95% CI)	CMR (95% CI)	*P*
Total	48 902	751		15.4 (14.3–16.5)	
Sex					<.001
Female	25 515	329	12.9 (11.6–14.4)		
Male	23 387	422	18.1 (16.4–19.9)		
Age (in 2004)					<.001
15–24 y	12 455	45	3.6 (2.6–4.8)		
25–36 y	12 741	82	6.4 (5.1–8.0)		
37–54 y	12 912	161	12.5 (10.6–14.6)		
55–90 y	10 794	463	42.9 (39.1–47.0)		
*L. loa* microfilaremia					.383
Negative	40 952	620	15.2 (13.9–16.4)		
Positive	7950	131	16.5 (13.8–19.6)		
MFD of *L. loa*					.603
0	40 952	620	15.2 (13.9–16.4)		
1–7999	6766	110	16.3 (13.7–19.6)		
8000–29 999	692	14	20.7 (11.1–34.0)		
≥30 000	492	7	14.2 (5.7–29.3)		
*M. perstans* microfilaremia					.231
Negative	44 265	684	15.5 (14.3–16.7)		
Positive	1898	36	19.0 (13.3–26.3)		
MD^a^	2739	31	11.4 (7.7–16.1)		
History of eyeworms					.010
Negative	26 315	369	14.0 (12.6–15.5)		
Positive	22 587	382	17.0 (15.3–18.7)		
Village environment					.922
Forest	14 122	212	15.0 (13.1–17.2)		
Mixed	15 286	242	15.8 (13.9–18.0)		
Savannah	19 494	297	15.2 (13.6–17.1)		
CMFL					.195
<0.4	13 539	191	14.1 (12.2–16.3)		
0.4–1.85	11 820	174	14.7 (12.7–17.1)		
1.86–3.84	12 512	226	18.1 (15.9–20.6)		
>3.84	11 006	160	14.5 (12.5–17.0)		

*P* values were estimated using the Mantel-Haenszel method.

Abbreviations: CMFL, community microfilarial load (expressed in mf per mL); CMR, crude mortality rate; MFD, microfilarial density (expressed in mf per mL); PY, person-years of follow-up; SMR, categories-specific mortality rate.

a Missing data; 187 out of 3329 (5.6%) individuals included in the analyses had missing data for the variable “*M. perstans.*”

The year and month of death could be determined for 404 of the 751 deceased (53.8%), while only the year could be determined for the other 347 (46.2%).

### Evaluation of Individual-Level Survival Between 2004 and 2021

There was a significant interaction between age and *L. loa* microfilaremic status (*P* < .001), with *L. loa*–positive status having an increased effect on mortality in younger individuals. No additional interactions were detected. As the analysis of Schoenfeld residuals led to rejection of the proportional hazard hypothesis for the variables *L. loa* microfilaremia, sex, age, and eyeworm history (*P* < .001, *P* = .015, *P* < .001, and *P* = .040, respectively), an AFT model was applied. The random effect at the village level was significant (*P* < .001). According to the AIC, we used a Weibull distribution to fit the model. Finally, as Cramer's V test showed a strong association (V > 0.30) between the village environment type and the CMFL, only the latter variable was included in the analyses. A time ratio (TR) variable was obtained from the AFT model with a Weibull distribution; a TR <1 means a reduced survival time—that is, an increased risk of mortality; and a TR >1 means an increased survival time—that is, a decreased risk of mortality.

In the final models, only age, sex, and *L. loa* microfilaremic status were significantly associated with mortality. The adjusted time ratio (aTR) for microfilaremic individuals was, at baseline, 0.42 (*P <* .001), meaning that, all other things being equal, the survival time of microfilaremic individuals was shorter by 58% when compared with that of amicrofilaremic individuals (Table 2). When we considered the *L. loa* MFD categorization, the aTR for subjects with initial MFDs of 1–259, 260–1799, and ≥1800 mf/mL were 0.43 (*P* = .002), 0.30 (*P* < .001), and 0.54 (*P* = .018), respectively, compared with the amicrofilaremics. Last, compared with the amicrofilaremic group, the aTRs were 0.43 (*P* < .001) and 0.36 (*P* = .030) in the groups with 1–9999 and ≥10 000 mf/mL, respectively.

**Table 2.** Relationship Between Mortality and Independent Variables at the Individual Level by Survival Model

	Saturated Multivariable Model		Final Multivariable Model	
	aTR (95% CI)	*P*	aTR (95% CI)	*P*
Age (continuous)	0.96 (0.96–0.97)	<.001	0.96 (0.96–0.97)	<.001
Age (continuous) * *L. loa* microfilaremia	1.02 (1.01–1.03)	<.001	1.02 (1.01–1.03)	<.001
*L. loa* microfilaremia (reference: negative)	0.42 (0.28–0.63)	<.001	0.42 (0.28–0.63)	<.001
Sex (reference: female)	0.72 (0.66–0.79)	<.001	0.72 (0.65–0.79)	<.001
*M. perstans* microfilaremia (reference: negative)				
Positive	1.02 (0.81–1.28)	.861		
MD^a^	1.12 (0.87–1.44)	.385		
History of eyeworms (reference: negative)	1.01 (0.91–1.12)	.877		
CMFL (reference <0.4)				
0.4–1.85	1.01 (0.84–1.21)	.911		
1.86–3.84	0.88 (0.74–1.06)	.190		
>3.84	1.03 (0.85–1.26)	.764		
Age (continuous)	0.96 (0.96–0.97)	<.001	0.96 (0.96–0.97)	<.001
Age (continuous) * *L. loa* microfilaremia (reference: negative)				
1–259	1.02 (1.01–1.03)	.003	1.02 (1.01–1.03)	.002
260–1799	1.03 (1.01–1.04)	<.001	1.03 (1.01–1.04)	<.001
>1799	1.01 (1.00–1.02)	.018	1.01 (1.00–1.02)	.018
*L. loa* microfilaremia (reference: negative)				
1–259	0.44 (0.22–0.86)	.017	0.43 (0.22–0.84)	.014
260–1799	0.30 (0.16–0.55)	<.001	0.30 (0.16–0.56)	<.001
>1799	0.54 (0.28–1.02)	.057	0.54 (0.29–1.01)	.056
Sex (reference: female)	0.72 (0.66–0.79)	<.001	0.72 (0.65–0.79)	<.001
*M. perstans* microfilaremia (reference: negative)				
Positive	1.01 (0.81–1.26)	.951		
MD^a^	1.12 (0.87–1.44)	.381		
History of eyeworms (reference: negative)	1.01 (0.91–1.12)	.909		
CMFL (reference <0.4)				
0.4–1.85	1.01 (0.84–1.21)	.913		
1.86–3.84	0.88 (0.74–1.06)	.184		
>3.84	1.04 (0.85–1.26)	.732		
Age (continuous)	0.96 (0.96–0.97)	<.001	0.96 (0.96–0.97)	<.001
Age (continuous) * *L. loa* microfilaremia (reference: negative)				
1–9999	1.02 (1.01–1.03)	<.001	1.02 (1.01–1.03)	<.001
>10 000	1.02 (1.00–1.04)	.013	1.02 (1.00–1.04)	.012
*L. loa* microfilaremia (reference: negative)				
1–9999	0.43 (0.28–0.66)	<.001	0.43 (0.28–0.66)	<.001
>10 000	0.35 (0.14–0.91)	.031	0.36 (0.14–0.91)	.031
Sex (reference: female)	0.72 (0.66–0.79)	<.001	0.72 (0.66–0.79)	<.001
*M. perstans* microfilaremia (reference: negative)				
Positive	1.02 (0.82–1.28)	.836		
MD^a^	1.12 (0.87–1.44)	.387		
History of eyeworms (reference: negative)	1.01 (0.91–1.12)	.865		
CMFL (reference <0.4)				
0.4–1.85	1.01 (0.84–1.21)	.916		
1.86–3.84	0.88 (0.74–1.06)	.189		
>3.84	1.03 (0.85–1.26)	.767		

Abbreviation: aTR, adjusted time ratio; CMFL, community microfilarial load (expressed in mf per mL); MD, missing data.

a Missing data (n = 187, 5.6%).

Overal median survival times provided by the AFT model were 55.4 (95% CI, 47.4–63.3) years for the whole study population, 58.5 (95% CI, 49.7–67.3) years for the amicrofilaremics, and 39.2 (95% CI, 32.6–45.8) years for the microfilaremics. For subjects with initial MFDs of 1–259, 260–1799, and ≥1800 mf/mL, median survival times were 40.2 (95% CI, 29.6–50.8), 38.4 (95% CI, 29.2–47.6), and 39.2 (95% CI, 28.5–49.9) years, respectively. Last, for subjects with initial MFDs of 1–9999 and ≥10 000 mf/mL, median survival times were 39.6 (95% CI, 32.5–46.7) and 36.5 (95% CI, 22.9–49.9) years, respectively.

**Figure 1.**
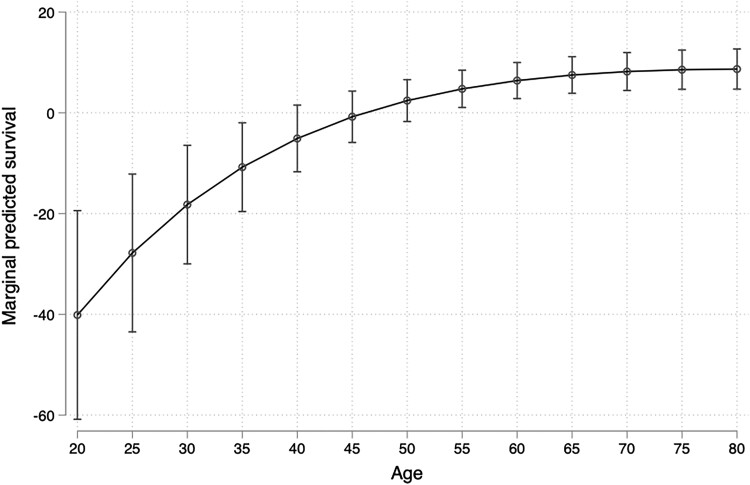
Predicted survival curve from the accelerated failure time model. Model using the *L. loa* microfilariae as binary illustrating the values of Table 3.

**Figure 2.**
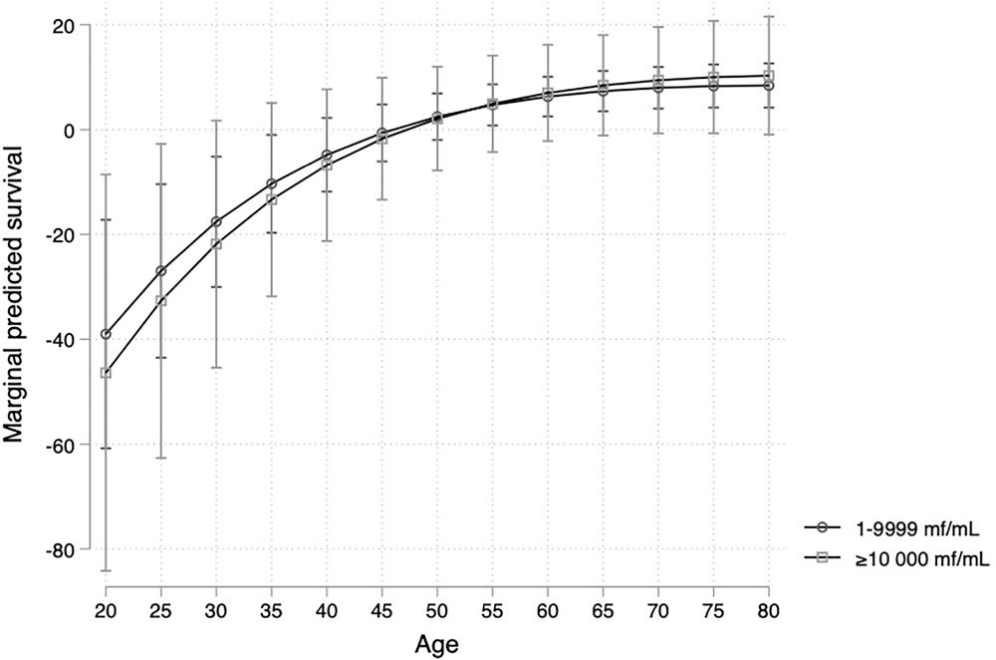
Predicted survival curve from the accelerated failure time model. Model using the *L. loa* microfilariae in categories (0; 1–9999; and ≥10 000 mf/mL) illustrating the values of Table 3.

Interaction terms between *L. loa* mf status and age were >1; that is, the excess mortality associated with *L. loa* microfilaremia decreases with age. Figures 1 and 2 show how the predicted effects of microfilaremia compared with amicrofilaremia vary according to the age. Detailed values are presented in Table 3. Up to the age of 35 years, microfilaremic individuals had a significantly reduced survival time when compared with the amicrofilaremics, and conversely microfilaremics aged >55 years had a slightly longer survival time than amicrofilaremics. The models also suggested a small effect of MFD. For instance, for individuals aged 20 years in 2004, the median survival times of those who harbored 1–9999 and >10 000 mf/mL at that time were reduced by 39.0 and 46.4 years, respectively, compared with the median survival time of the amicrofilaremics. After the age of 35, having an MFD >10 000 mf/mL was not associated with a reduced survival time.

**Table 3.** Predicted Survival at 20, 25, 30, 35, 40, 45, 50, 55, 60, 65, 70, 75, and 80 Years Old (Reference Category: Amicrofilaremia); for Each Age, Values Indicate the Difference of Predicted Median Survival Times in the Microfilaremic Population Compared With the Amicrofilaremic Population

	Binary Model		Model With 3 Categories of *L. loa* MFD						Model with 2 Categories of *L. loa* MFD			
Age	Positive	*P*	1–259 mf/mL	*P*	260–1799 mf/mL	*P*	≥1800 mf/mL	*P*	1–9999 mf/mL	*P*	≥10 000 mf/mL	*P*
20	−40.1 (−60.8; −19.4)	<.001	−38.4 (−69.7; −7.1)	.016	−50.9 (−76.1; −25.7)	<.001	−30.9 (−63.6; 1.9)	.065	−39.0 (−60.8; −17.3)	<.001	−46.4 (−84.2; −8.6)	.016
25	−27.8 (−43.5; −12.2)	<.001	−26.1 (−50.4; −1.8)	.035	−35.9 (−55.5; −16.2)	<.001	−21.3 (−46.5; 3.8)	.097	−27.0 (−43.5; −10.5)	.001	−32.7 (−62.6; −2.7)	.033
30	−18.2 (−30.0; −6.5)	.002	−16.6 (−35.2; 2.1)	.082	−23.9 (−39.2; −8.7)	.002	−14.0 (−33.1; 5.2)	.153	−17.6 (−30.1; −5.1)	.006	−21.8 (−45.4; −1.7)	.049
35	−10.8 (−19.6; −2.0)	.016	−9.2 (−23.4; 5.1)	.208	−14.5 (−26.3; 2.6)	.017	−8.4 (−22.8; 6.1)	.255	−10.3 (−19.7; −1.0)	.030	−13,4 (−31.8; 5.1)	.155
40	−5.1 (−11.7; −1.5)	.031	−3.5 (−14.5; 7.4)	.527	−7.1 (−16.4; 2.2)	.137	−4.2 (−15.0; 6.7)	.451	−4.8 (−11.9; 2.2)	.181	−6.8 (−21.3; 7.7)	.358
45	−0.8 (−5.9; 4.3)	.761	0.7 (−7.9; 9.3)	.869	−1.3 (−9.0; 6.3)	.734	−1.0 (−9.2; 7.1)	.803	−0.6 (−6.1; 4.8)	.820	−1.7 (−13.4; 9.9)	.769
50	2.4 (−1.7; 6.6)	.255	3.9 (−3.3; 11.0)	.291	3.1 (−3.7; 9.9)	.372	1.2 (−5.1; 7.6)	.705	2.5 (−1.9; 6.9)	.273	2.1 (−7.8; 12.0)	.681
55	4.7 (1.0; 8.5)	.012	6.1 (−0.4; 12.7)	.068	6.5 (−0.2; 13.1)	.056	2.8 (−2.4; 8.1)	.292	4.7 (0.8; 8.6)	.018	4.9 (−4.3; 14.1)	.293
60	6.4 (2.8; 10.0)	<.001	7.7 (1.2; 14.3)	.021	9.0 (2.1; 15.8)	.011	3.9 (−0.8; 8.7)	.105	6.3 (2.5; 10.1)	.001	7.0 (−2.2; 16.2)	.135
65	7.5 (3.9; 11.1)	<.001	8.7 (1.9; 15.5)	.012	10.8 (3.4; 18.1)	.004	4.6 (0.0; 9.2)	.048	7.3 (3.5; 11.2)	<.001	8.5 (−1.1; 18.0)	.084
70	8.2 (4.4; 12.0)	<.001	9.4 (2.2; 16.5)	.010	12.0 (4.1; 20.0)	.003	5.0 (0.4; 9.6)	.032	8.0 (4.0; 12.0)	<.001	9.4 (−0.7; 19.6)	.069
75	8.6 (4.7; 12.4)	<.001	9.7 (2.2; 17.2-	.011	12.8 (4.3; 21.4)	.003	5.2 (0.6; 9.8)	.028	8.3 (4.2; 12.4)	<.001	10.0 (−0.7; 20.7)	.067
80	8.7 (4.7; 12.7)	<.001	9.7 (2.0; 17.5)	.014	13.3 (4.3; 22.3)	.004	5.2 (0.5; 9.9)	.029	8.4 (4.2; 12.6)	<.001	10.3 (−0.9; 21.5)	.072

Abbreviation: MFD, microfilarial density.

### Sensitivity Analyses

When all LFTUs are excluded from the analyses, overall median survival times were 55.5 (95% CI, 47.1–63.8) and 37.7 (95% CI, 31.4–44.0) years for the amicrofilaremic and microfilaremic populations, respectively. For subjects with initial MFDs of 1–259, 260–1799, and ≥1800 mf/mL, median survival times were 37.4 (95% CI, 27.7–47.0), 37.3 (95% CI, 28.4–46.1), and 38.9 (95% CI, 28.2–49.7) years, respectively. In addition, for subjects with initial MFDs of 1–9999 and ≥10 000 mf/mL, median survival times were 37.9 (95% CI, 31.2–44.6) and 36.4 (95% CI, 22.7–49.9) years, respectively.

Finally, in our study population, from our population-level model, average survival times were 55.4 (95% CI, 46.0–64.7) years in the amicrofilaremics and 36.9 years in the microfilaremics, that is, 18.5 years earlier (95% CI, 2.7–34.2; *P* = .021). In addition, after having excluded all LFTUs, microfilaremics would have died on average 17.8 years earlier (*P* = .016) than the amicrofilaremics.

## DISCUSSION

This study aimed to evaluate the effect of loiasis on the survival duration of a population aged ≥15 years living in an endemic area of the Republic of Congo. We estimated that the median and average survival times were 19.3 and 18.5 (*P* = .021) years less in the microfilaremics than in amicrofilaremics. Individual-level survival analyses showed that the excess mortality was associated with *L. loa* microfilaremia, with the magnitude of this excess mortality being greatest in younger individuals.

Despite the long follow-up period, the proportion of LTFUs was relatively low (13.2%), indicating a stable population between 2004 and 2021. During this period, no CDTI was administered, suggesting that the cohort remained exposed to a constant force of infection for *L. loa*. *L. loa* MFDs in microfilaremic individuals have previously been shown to remain fairly stable over several months or years [17–19]. It is therefore unlikely that individuals who were microfilaremic in 2004 became amicrofilaremic during the follow-up period. However, it is more than likely that a proportion of those found to be amicrofilaremic in 2004 became microfilaremic thereafter, which may be a limitation to our study and interpretation.

The differential age effect found in the analysis may be explained by a survival bias. This hypothesis potentially implies that some pathophysiological mechanisms (immunological or inflammatory) would arise during the first years of microfilaria acquisition, before MFD becomes stable, and that individuals who have survived this phase are no longer at risk of excess mortality.

Although our analyses highlight an effect of microfilaremic status on survival, we did not identify a clear relationship between MFD level and mortality risk and identified significant differences in survival time only across some age categories. Important caveats to the presented results therefore include the potential of other differences between the microfilaremic and amicrofilaremic populations that have not been measured here (such as socioeconomic status, other infections, etc.), and which may therefore (in part) confound our results. However, as a lack of power may also explain these results, new cohort studies, as well as further analyses pooling cohorts from Cameroon and Congo, should be conducted to get more accurate estimations. Although the methodology (environment and individuals selected, questionnaire, parasitological assessment, and level of microfilarial individuals’ densities) was the same in Cameroon and Congo, and the number of PY was close (45 164 and 48 902, respectively), observed crude mortality rates were slightly higher in Cameroon (20.3 and 15.36 per 1000 PY, respectively, in Cameroon and in Congo). Other factors that were not controlled for in these analyses and that differed between the 2 regions may explain these different crude mortality rates. This new study provides new evidence that survival is impacted not only in individuals with high microfilarial densities, as concluded in the previous paper [13], but across the whole population of microfilaremic individuals. The absence of such an effect in the previous study is likely a consequence of a lack of statistical power. Finally, we observed that the effect of loiasis on mortality changed according to the age of the individuals—we currently lack an explanation for this phenomenon. This result should be considered when developing protocols for further studies intended to understand the physiopathological mechanisms associated with loiasis.

The fact that a history of eyeworm episodes was not found to be associated with excess mortality suggests that the latter is due to the microfilaremia and not to the presence of adult *L. loa.* The persistent presence of *L. loa* mf in the bloodstream and in the organs could induce chronic pathogenic mechanisms, leading to early death. These mechanisms could include obstructive or inflammatory processes, as demonstrated in the retinal vessels [20–23], or indirect immunologically mediated phenomena, inducing pathogenic processes in various organs. High MFDs could also lead to a specific immunological status or to interactions with other pathogens, facilitating the development of processes not directly due to *L. loa* [24]. It should be noted that the immunological profile differs between microfilaremic and amicrofilaremic subjects [25, 26].

The main limitations of this study are its retrospective aspect and the problem of possible transitions (from amicrofilaremic to microfilaremic status, and vice versa) of the “exposure” factor leading to difficulties in interpreting the results. Indeed, prospective observational studies are imperative to better identify possible health events directly or indirectly related to excess mortality in the population.

The results of this retrospective cohort study confirm those obtained in Cameroon [13]. This further evidence of the severity of the disease [2, 9, 11–13] should contribute to its inclusion in the World Health Organization’s list of Neglected Tropical Diseases and lead to increased research into safe and effective treatments and the implementation of appropriate strategies to control loiasis in endemic populations.

## Supplementary Material

ofad103_Supplementary_Data

## Supplementary Data

Supplementary materials are available at *Open Forum Infectious Diseases* online. Consisting of data provided by the authors to benefit the reader, the posted materials are not copyedited and are the sole responsibility of the authors, so questions or comments should be addressed to the corresponding author.
