# Supplementary material for: Excess Mortality Associated With Loiasis: Confirmation by a New Retrospective Cohort Study Conducted in the Republic of Congo
Source: Open Forum Infect Dis. 2023 Feb 24;10(3):ofad103. doi: 10.1093/ofid/ofad103 (PMC10034755; doi:10.1093/ofid/ofad103)
Supplement: ofad103_Supplementary_Data [file ofad103_supplementary_data.docx]

| **Village** | **Participants in 2004** | **Retrieved in 2021** | **Prevalence *Loa* ≥ 8000 mf/mL** | **Prevalence *Loa* ≥ 30 000 mf/mL** | | **Prevalence *Loa***  **mf in 2004** | ***Loa* CMFL**  **in 2004** | **Prevalence *Loa***  **mf in 2021** | ***Loa* CMFL^*^**  **in 2021** |
| --- | --- | --- | --- | --- | --- | --- | --- | --- | --- |
|  | n | n | (%) | (%) | (%) | | mf/mL | (%) | mf/mL |
| Komono Centre | 85 | 69 | 2.4 | 2.4 | 18.8 | | 2.7 | 23.2 | 3.9 |
| Loyo | 68 | 59 | 5.9 | 0 | 23.5 | | 3.8 | 27.1 | 5.1 |
| Makaga | 78 | 78 | 2.6 | 0 | 33.3 | | 6.7 | 33.3 | 6.7 |
| Mambouana | 84 | 71 | 3.6 | 3.6 | 25.0 | | 5.4 | 29.6 | 8.0 |
| Mapati | 61 | 59 | 1.6 | 0 | 31.1 | | 7.0 | 28.8 | 6.3 |
| Mayeyé | 47 | 46 | 4.3 | 0 | 42.6 | | 9.9 | 43.5 | 10.5 |
| Mbaka | 53 | 43 | 1.9 | 0 | 7.5 | | 0.9 | 7.0 | 0.86 |
| Mbaya | 82 | 82 | 1.2 | 0 | 17.1 | | 2.2 | 17.1 | 2.2 |
| Mbila | 85 | 84 | 0 | 0 | 25.9 | | 3.3 | 26.2 | 3.3 |
| Mikakaya | 61 | 59 | 8.2 | 3.3 | 50.8 | | 31.7 | 52.5 | 35.8 |
| Minguelé | 49 | 47 | 2.0 | 0 | 34.7 | | 5.5 | 34.0 | 5.3 |
| Moetché | 28 | 25 | 7.1 | 7.1 | 21.4 | | 6.5 | 20.0 | 5.9 |
| Moutouala | 32 | 32 | 3.1 | 0 | 25.0 | | 3.5 | 25.0 | 3.5 |
| Mvakala | 70 | 68 | 7.1 | 2.9 | 32.9 | | 12.0 | 33.8 | 13.0 |
| Ouandzi | 54 | 51 | 1.9 | 3.7 | 25.9 | | 5.3 | 27.5 | 6.0 |

**Supplementary Table 1. Summary of results obtained in 2004 and number of individuals found in 2021, by village in the forest zone.**

**^*^** CMFL: community microfilarial load (expressed in mf per mL).

Prevalence *Loa* ≥ 8000 mf/ml = proportion of subjects with ≥ 8000 *Loa* mf/mL among all those examined in the village in 2004.

Prevalence *Loa* ≥ 30 000 mf/ml = proportion of subjects with ≥ 30 000 *Loa* mf/mL among all those examined in the village in 2004.

Prevalence *Loa* mf in 2004 = proportion of *Loa* mf carriers among all those examined in the village in 2004.

Prevalence *Loa* mf in 2021 = proportion of *Loa* mf carriers in 2004 among all subjects retrieved in 2021.

| **Village** | **Participants in 2004** | **Retrieved in 2021** | **Prevalence *Loa***  **≥ 8000 mf/mL** | **Prevalence *Loa* ≥ 30 000 mf/mL** | **Prevalence *Loa***  **mf in 2004** | ***Loa* CMFL**  **in 2004** | **Prevalence *Loa***  **mf in 2021** | ***Loa* CMFL**  **in 2021** |
| --- | --- | --- | --- | --- | --- | --- | --- | --- |
|  | n | n | (%) | (%) | (%) | mf/mL | (%) | mf/mL |
| Bihoua | 84 | 55 | 3.6 | 1.2 | 28.6 | 5.8 | 25.5 | 5.6 |
| Congo Malembé | 48 | 45 | 0 | 2.1 | 16.7 | 2.0 | 17.8 | 2.2 |
| Indo | 102 | 57 | 0 | 0 | 19.6 | 2.4 | 17.5 | 2.1 |
| Kihouba | 23 | 20 | 0 | 0 | 4.4 | 0.1 | 0 | 0 |
| Kikondé | 84 | 80 | 0 | 0 | 17.9 | 1.9 | 18.8 | 2.0 |
| Kimpolo | 98 | 85 | 0 | 3.1 | 7.1 | 0.8 | 7.1 | 0.7 |
| Kitaka | 33 | 31 | 0 | 0 | 0 | 0 | 0 | 0 |
| Kolo | 81 | 67 | 0 | 0 | 22.2 | 3.3 | 23.9 | 4.1 |
| Kouanga | 45 | 36 | 2.2 | 2.2 | 20.0 | 3.5 | 19.4 | 3.4 |
| Lissiemi | 82 | 75 | 1.2 | 0 | 10.9 | 1.0 | 12.0 | 1.8 |
| Makoubi | 85 | 73 | 1.17 | 1.2 | 10.6 | 1.0 | 10.9 | 0.9 |
| Manengué | 13 | 4 | 0 | 7.7 | 23.1 | 5.6 | 75.0 | 8.7 |
| Mbomo II | 19 | 17 | 0 | 0 | 0 | 0 | 0 | 0 |
| Mikamba | 91 | 66 | 3.2 | 0 | 26.4 | 3.8 | 25.8 | 4.0 |
| Mouindi | 57 | 43 | 0 | 0 | 0 | 0 | 0 | 0 |
| Moumanga | 54 | 48 | 0 | 0 | 14.8 | 1.6 | 16.7 | 1.9 |
| Moussehou | 57 | 39 | 1.8 | 0 | 15.8 | 1.8 | 23.1 | 3.5 |
| Moussoki Mpé | 8 | 7 | 0 | 0 | 12.5 | 1.2 | 0 | 0 |
| Ossiba | 66 | 47 | 3.0 | 1.5 | 40.9 | 14.6 | 46.8 | 24.3 |
| Pika Songo | 75 | 64 | 0 | 0 | 4.0 | 0.4 | 4.7 | 0.4 |
| Pini | 80 | 49 | 1.3 | 0 | 25.0 | 2.8 | 32.7 | 5.2 |
| Soulou | 86 | 84 | 0 | 0 | 2.3 | 0.1 | 1.2 | 0.04 |

**Supplementary Table 2. Summary of results obtained in 2004 and number of individuals found in 2021 per village in the mixed zone.**

| **Village** | **Participants in 2004** | **Retrieved in 2021** | **Prevalence *Loa* ≥ 8000 mf/mL** | **Prevalence *Loa* ≥ 30000 mf/mlL** | **Prevalence *Loa***  **mf in 2004** | ***Loa* CMFL**  **in 2004** | **Prevalence *Loa***  **mf in 2021** | ***Loa* CMFL**  **in 2021** |
| --- | --- | --- | --- | --- | --- | --- | --- | --- |
|  | n | n | (%) | (%) | (%) | mf/mL | (%) | mf/mL |
| Diamboufouana | 27 | 24 | 0 | 0 | 3.7 | 0.4 | 4.2 | 0.4 |
| Dibeni | 54 | 50 | 0 | 1.8 | 7.4 | 0.8 | 6.0 | 0.6 |
| Ditadi | 60 | 53 | 0 | 0 | 3.3 | 0.3 | 3.8 | 0.3 |
| Iloupanga | 90 | 81 | 2.2 | 0 | 16.7 | 2.0 | 17.3 | 2.2 |
| Kikanou | 60 | 58 | 0 | 0 | 3.3 | 0.2 | 1.7 | 0 |
| Kikassa | 85 | 74 | 0 | 0 | 2.4 | 0.2 | 2.7 | 0.2 |
| Lissanga | 50 | 40 | 0 | 0 | 12.0 | 1.1 | 10.0 | 0.8 |
| Louvakou centre | 85 | 74 | 0 | 0 | 5.9 | 0.4 | 5.4 | 0.4 |
| Mafoubou | 50 | 44 | 0 | 0 | 4.0 | 0.4 | 4.5 | 0.5 |
| Malolo | 60 | 56 | 0 | 0 | 3.3 | 0.2 | 1.8 | 0.1 |
| Mangandzi | 73 | 73 | 0 | 1.4 | 4.1 | 0.4 | 4.1 | 0.4 |
| Mont Belo | 55 | 49 | 0 | 0 | 3.6 | 0.3 | 2.0 | 0.2 |
| Moukondo | 48 | 46 | 0 | 0 | 20.8 | 4.1 | 21.7 | 4.4 |
| Ndembo | 51 | 47 | 0 | 8.3 | 1.9 | 0.2 | 2.1 | 0.2 |
| Tao Tao | 90 | 85 | 1 | 2.0 | 6.7 | 0.8 | 7.1 | 0.8 |
| Yandza | 83 | 70 | 0 | 0 | 3.6 | 0.2 | 4.3 | 0.3 |

**Supplementary Table 3. Summary of results obtained in 2004 and individuals recovered in 2021, by village in the savannah zone.**

|  | **Participants** | **Microfilaremics** | **Prevalence** |  |  |
| --- | --- | --- | --- | --- | --- |
|  | n n % (95 CI) | | | n | % (95% CI) |
| Sex |  |  |  |  |  |
| Females | 1710 | 208 | 12.2 (10.6-13.9) |  |  |
| Males | 1619 | 332 | 20.5 (18.4-22.8) |  |  |
| Age (years) in 2004 |  | | |  |  |
| 15-19 | 374 | 25 | 6.7 (4.3-9.9) |  |  |
| 20-29 | 758 | 102 | 13.5 (11.0-16.3) |  |  |
| 30-39 | 621 | 123 | 19.8 (16.5-23.6) |  |  |
| 40-49 | 479 | 82 | 17.1 (13.6-21.2) |  |  |
| 50-59 | 436 | 77 | 17.7 (13.9-22.1) |  |  |
| 60-90 | 661 | 131 | 19.8 (16.6-23.5) |  |  |
| Environment |  |  |  |  |  |
| Savannah | 1021 | 66 | 6.5 (4.9-8.2) |  |  |
| Mixed | 1371 | 217 | 15.8 (13.8-18.1) |  |  |
| Forest | 937 | 257 | 27.4 (24.2-30.9) |  |  |
| Total | 3329 | 540 | 16.2 (14.9-17.6) |  |  |

**Supplementary Table 4. Summary of parasitological results obtained in 2004 according to sex, age, and environment type in the village of residence.**

**Population-level survival analysis using an average treatment effect (ATE) model**

In addition of these individual analyses, we conducted an ATE analysis, which is an analysis suited to analyze observational cohort data where, by definition, the main exposure variable (here, the *L. loa* microfilaremic status) cannot be randomised.

Among the possible ATE models, we applied a model that was composed of three parts (or sub-models). This model aimed to explain survival (outcome sub-model) while considering the factors determining the main exposure and integrating censoring (censoring sub-model). This model is called the “outcome and treatment model” and was carried out with the command *stteffects ipwra* in STATA.

The survival sub-model (outcome sub-model) was also adjusted for age and age*age, sex, *M. perstans* microfilaremia, eyeworm history and CMFL in the village of residence. A Weibull distribution was applied for the survival sub-model. The treatment sub-model used a logit link and an inverse probability weighted covariate adjustment.

According to the recommended procedure of *stteffects ipwra*, the choice of variables included in the treatment sub-model was assessed using a *Chi-2* test [1–2]. This uses a propension score to obtain balanced covariables explaining our main exposure (*Loa loa* negative or positive). According to the procedure to have balanced covariables, we included the variables gender, CMFL and age (and age*age) in the treatment model.

ATE analyses produce estimates of an an average effect of the main exposure variable in the study population. We estimated the mean difference in time to death between (a) a situation where everyone would have been microfilaremic for *L. loa* and (b) a situation where no one would have been microfilaremic.

1. Lin DY. MULCOX2: A general computer program for the Cox regression analysis of multivariate failure time data. Comput Methods Programs Biomed 1993; 40:279–93.

2. Lin DY. Cox regression analysis of multivariate failure time data: the marginal approach. Stat Med 1994; 13:2233–47.

3. Rondeau V, Commenges D, Bourdel-Marchasson I. Etude multicentrique de la survenue d’escarres par un modèle de Cox basé sur une approche marginale. Rev Epidemiol Sante Publique 1999; 47:555–61.
